# Response to the letter to the editor

**DOI:** 10.1007/s00784-026-06823-w

**Published:** 2026-03-25

**Authors:** Marco Bonilla, Francisco Mesa

**Affiliations:** Department of Periodontics, School of Dentistry, University of Granada, Granada, 18071 Spain

## Response to the letter to the editor

After carefully reviewing the Letter to the Editor authored by Dr. Osorio [1], we would like to provide clarification on several of the points raised.

- She states that the conclusion of the study: *“…However*,* the absence of an association with other histopathological variables suggests that periodontitis is not related to cancer severity”* cannot be drawn from a cross-sectional study.

The design of our study was observational, cross-sectional, with two comparative groups, in which it is appropriate to analyze risk variables and determinants that influence the probability of suffering another disease. In this sense, periodontitis behaves as such by showing a strong association with CEA, while it does not do so with any of the other 11 histopathological-morphometric variables analyzed [2]. The terms “favor” and “influence” used are intended to reflect this added risk. Cross-sectional studies assess exposure and disease simultaneously in a well-defined population at a specific point in time. Regardless of the level of evidence and the fact that each study design has its advantages and limitations, making them suitable for different scenarios, in the design proposed in this paper a control group is not necessary. In our study, two groups of patients with CRC were compared – those with periodontitis and those without periodontitis – rendering a separate healthy control group unnecessary. As described in the objectives of the study, in a population with CRC, the aim is to assess whether periodontitis is associated with specific tumor characteristics. This is a valid design to explore intra-disease associations and pathophysiological mechanisms in this population.

- She describes our result of a 69.5% prevalence of periodontitis in the CRC patient group as a “supposition” and as “inconsistent.”

Precisely, if there is an epidemiological design suitable for determining prevalence, it is the cross-sectional design. For this result, we do acknowledge as a limitation – and this is stated in the discussion section of the manuscript (page 7, first paragraph) [2] – that we did not determine the prevalence of periodontitis in a matched control group from the same universe as the CRC group. Instead, we provided a prevalence figure for periodontitis in the general population, updated and as valid as any other (WHO Data Bank for Spain) [3], with the intention of demonstrating to the reader the magnitude of the finding. Subsequently, Dr. Osorio provides two bibliographic references to argue that this prevalence result, from “her personal opinion,” does not represent an increased figure. The study by Morales A. et al. [4] compared the prevalence of periodontitis between the AAP/EFP and CDC/AAP classification systems and evaluated the accuracy of the new AAP/EFP classification system against the CDC/AAP case definition for population-based studies. It revealed a clear discrepancy in periodontitis prevalence between the AAP/EFP and CDC/AAP classifications when using epidemiological data; therefore, rebutting prevalence data across different sources does not make sense.

It is surprising that, solely due to the lack of coincidence in prevalence figures, it is stated that the rest of the analytical association results would not exist and again describes the study as having an “evident lack of quality”.

- She states that training and calibration in periodontal research should preferably be carried out not by a reference investigator, but by an experienced WHO epidemiologist, using a specifically designed calibration program.

The WHO recommendation cited refers specifically to basic oral health population surveys (“basic oral health surveys”) with multiple examiners [5]. In those contexts, standardization among several teams is crucial, and therefore an experienced epidemiologist is suggested as a validator. This has nothing to do with the present study. Therefore, the reference used in the letter, we believe, is not appropriate; perhaps she intended to refer to: *WHO. Calibration of examiners for oral health epidemiological surveys. Geneva*,* WHO*,* 1993* [6]. The essence of calibration is to have a gold standard or reference examiner against whom the clinical assessment of the examiners (usually several) must be contrasted in the case of large epidemiological surveys. Our article is not a survey; there is a single examiner, and methodologically calibration must be performed against a reference examiner. Therefore, we do not understand the criticism.

- She states that it is essential that periodontal recording and diagnosis be performed by a dentist; otherwise, this may directly affect the quality of the periodontal variables recorded, which are key to the results and conclusions of the study.

We completely agree with this observation. In this regard, we would like to emphasize that E.L. (Esperanza Lanzas), as she is mentioned in the Materials and Methods section, is a licensed dentist with postgraduate training in periodontology and maintains an exclusive private practice in periodontology and oral surgery in Murcia, Spain.

- Dr. Osorio assumes that because both groups have a similar mean number of remaining teeth, there would be a diagnostic error in periodontitis, and she again qualifies the results as unreliable based on this fact.

Honestly, we do not understand why patients with periodontitis must necessarily have fewer teeth. Claiming that there is an error in the periodontal diagnosis based on this assumption does seem to us an inconsistency and a lack of scientific rigor.

- On the one hand, the letter’s author acknowledges that CEA is a phenotypic biomarker of the field of cancerization in CRC and provides a citation, only to then categorically state: *“it has never been shown how the presence or the upregulation of CEA increases the local risk of additional primary tumors or recurrences.”*

CEA is indeed a serological biomarker associated with an increased risk of recurrence, as previously demonstrated both in publications (Khan et al.: *“In patients with raised preoperative CEA*,* the risk of recurrence was 5.26-fold greater as compared to those with normal CEA levels (p = 0.028)”* [7]) and clinically, with monitoring of this biomarker being routine in the postoperative follow-up of these patients. We wish to state that three of the authors of this manuscript are medical specialists in Digestive Diseases, some with more than 35 years of professional activity.

It is possible that she has not understood the objectives of our article, nor does she seem to understand what we mean by periodontitis as a field of cancerization. It is not only the association with CEA, but, as explained in Fig. 1 of our manuscript [2], a combined effect of overexpression of IL-6, CEA, and *F. nucleatum* that disrupt gut microbiota homeostasis, alter anoikis and gene expression, and promote a pro-inflammatory CEA-producing state in the gut, fostering a tumor-promoting microenvironment.

- Dr. Osorio argues that: *“In 2003*,* it was published that salivary CEA levels in patients with periodontitis was significantly higher than those encountered in healthy control subjects. Salivary CEA concentrations also correlated with the stage of periodontitis*,* and the administration of metronidazole leaded to statistically significant decrease in salivary CEA concentration in all examined subjects.”*

However, the study to which she refers [8] concerns patients with acute necrotizing ulcerative periodontitis (NUP), a periodontal emergency that has nothing to do with the chronic periodontitis diagnosed in CRC patients. Using a study based on an acute pathology to refute CEA figures from our study, which is based on a chronic pathology, does not seem methodologically appropriate to us.

- In another paragraph of her letter, and with regard to CEA, Dr. Osorio states: *“in the study of Mesa-López et al.*,* the encountered elevated CEA levels in patients suffering CRC and periodontitis*,* if compared to those CRC patients without periodontitis*,* might just be related to the periodontal and systemic inflammation since proinflammatory mediators*,* present in periodontitis*,* increase the expression of CEA”.*

This is precisely one of the guiding threads of the discussion, on which we base our explanation of the results.

- Dr. Osorio states that the CEA values appear extremely low in our study.

As stated in the header of Table 2 of our manuscript [2], it is described that the values are expressed on a Log10 scale. The explanation for this statistical technique is also stated in the table footnote, *“Given the skewness of the distributions.”* To dispel any doubts, a new Table 2 with absolute values is attached to this letter, where the difference in CEA values in ng/mL between both groups can be verified.

- The author insists, with regard to CEA: *“No association should be stated without a previous statistical analysis about the influence of smoking as a confounding factor for serum CEA concentration values.”*

As shown in Table 1 of our manuscript [2], the smoking variable is similarly distributed between both groups, with a bivariate analysis yielding a p value of 0.136, indicating no statistically significant differences between them. It should be taken into account that, for a variable to be considered a confounder in the relationship between variable A and variable B, it must be associated with A, associated with B, and not be an intermediate causal link between them. Furthermore, in a study with a limited sample size, the investigator must be cautious regarding which variables are used for adjustment, as it would not be possible to adjust simultaneously for multiple variables. Adjustment was performed for age, as indicated in the second paragraph of the Results section, and no different results were obtained; therefore, these results are not shown.

- Once again, the results of the CA19-9 biomarker are misinterpreted. It is stated: “*In the present study by Mesa-López et al.*,* all patients had very low values of the CA19-9 marker*,* and in all cases these values were within the normal range. No explanation is provided for the rare low values of the analyzed serum markers.”*

We reiterate that the values are expressed on a Log10 scale. They are not very low values (a table with values in ng/ml is attached), and the corresponding explanation is indeed provided in the footnote of Table 2 (“Given the skewness of the distributions”).

We believe that the responses provided in the present letter help to clarify and address the concerns raised in the Letter to the Editor published in the Debate section.

P.S. Table 2 is attached, with values in ng/ml of the CEA and CA19-9 markers.

**Table 2:** Original: CEA (without log transformation) (*n* = 59)

Variable	Non periodontal mean ± sd	Periodontal mean ± sd	*P*-value^b^
Baseline CEA	1.7 ± 1.6	4.6 ± 7.9	< 0.001
Peak CEA	4.9 ± 10.5	13.2 ± 37.1	0.005
CA 19 − 9	21.2 ± 19.3	62.0 ± 194.91	0.926

b: Mann-Whitney test

## Data Availability

No datasets were generated or analysed during the current study.

## References

[1] Osorio R (2026) Letter to the editor. Clin Oral Investig 30:35. 10.1007/s00784-025-06707-5 41483026 10.1007/s00784-025-06707-5

[2] Mesa-López MJ, Bravo M, Egea J, El-Amrani S, Bonilla M, Alberca F, Mesa F (2025) Periodontitis as a field of cancerization: association with carcinoembryonic antigen in colorectal cancer patients. Clin Oral Investig 29:323. 10.1007/s00784-025-06399-x 40451975 10.1007/s00784-025-06399-x PMC12127229

[3] Nazir M, Al-Ansari A, Al-Khalifa K, Alhareky M, Gaffar B, Almas K (2020) Global prevalence of periodontal disease and lack of its surveillance. ScientificWorldJournal 2020:2146160. 10.1155/2020/2146160 10.1155/2020/2146160 PMC7275199 32549797

[4] Morales A, Strauss FJ, Hämmerle CHF, Romandini M, Cavalla F, Baeza M, Sanz M, Gamonal J (2022) Performance of the 2017 AAP/EFP case definition compared with the CDC/AAP definition in population-based studies. J Periodontol 93:1003–1013. 10.1002/JPER.21-0276 34625960 10.1002/JPER.21-0276

[5] World Health Organization (2013) Oral health surveys: basic methods, 5th edn. World Health Organization, Geneva

[6] World Health Organization (1993) Calibration of examiners for oral health epidemiological surveys. World Health Organization, Geneva

[7] Khan MA, Maken RN, Nisar H, Fatima I, Khan IU, Masood M, Shahid AB (2020) The role of preoperative carcinoembryonic antigen in recurrence of resectable colorectal carcinoma. Acta Clin Croat 59:216–222. 10.20471/acc.2020.59.02.03 33456107 10.20471/acc.2020.59.02.03 PMC7808221

[8] Golubovic S, Jankovic LJ, Movsesijan A, Bojic Z, Jankovic M (2003) Salivary carcinoembryonic antigen as an inflammatory marker. Jugoslov Med Biohem 22:207–211

